# Hemichorea-hemiballism: the role of imaging in diagnosing an unusual disorder in patients with nonketotic hyperglycemia

**DOI:** 10.1590/0100-3984.2015.0037

**Published:** 2016

**Authors:** Felipe Welter Langer, Gustavo Suertegaray, Daiane dos Santos, Giordano Rafael Tronco Alves, Carlos Jesus Pereira Haygert

**Affiliations:** 1 Hospital Universitário de Santa Maria (HUSM) - Universidade Federal de Santa Maria (UFSM), Santa Maria, RS, Brazil

**Figure 1:**
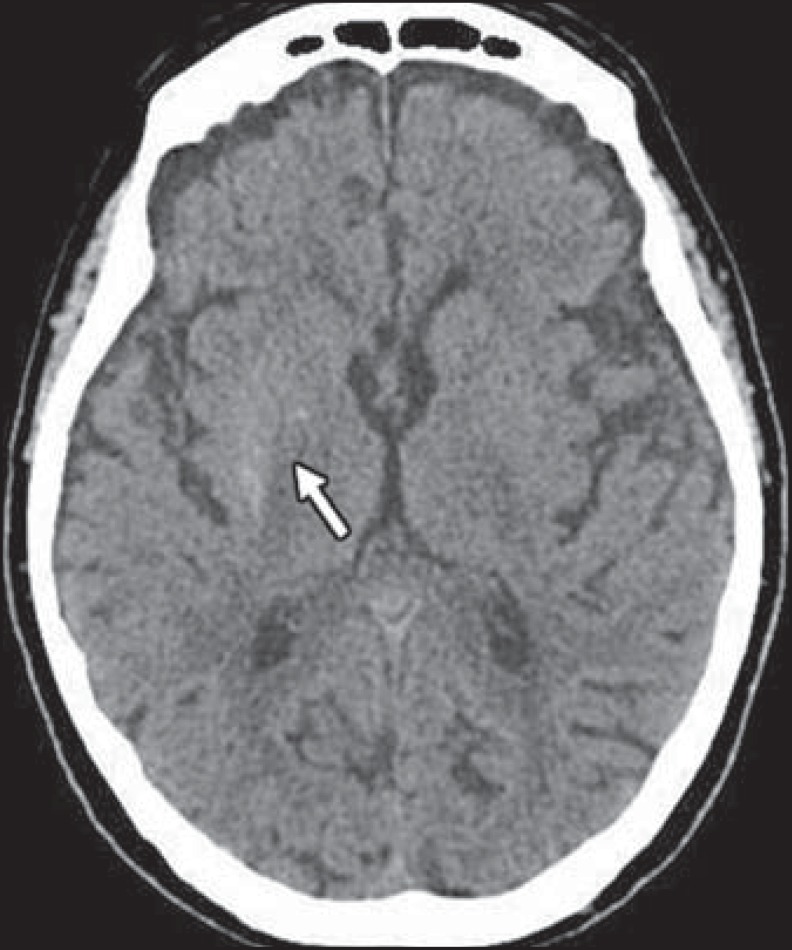
Axial unenhanced brain CT scan, acquired at hospital admission, showing right-sided hyperdensity in the putamen (arrow).

**Figure 2:**
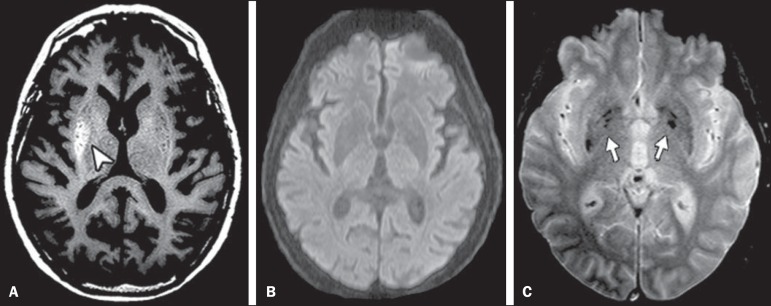
MRI findings two weeks after the initial presentation. **A:** Unenhanced T1-weighted spin-echo sequence showing a hyperintense lesion in the right putamen (arrowhead). **B:** Diffusion-weighted imaging sequence showing no restriction. **C:** T2*-weighted imaging showing bilateral hypointensities, presumably due to physiologic calcifications (arrows), in the globus pallidus.

*Dear Editor*,

An 81-year-old man presented to the emergency room with a 4-day history of progressive confusion followed by frontal headache and left-sided choreiform movements. His medical history was remarkable for smoking, dyslipidemia, and poorly-controlled hypertension, with no previous diagnosis of diabetes mellitus (DM). On laboratory investigation, his serum glucose was 460 mg/dL and his glycated hemoglobin was 17.4% (consistent with a prolonged period of undiagnosed DM). A computed tomography scan of the brain revealed hyperdensity of the right putamen without an associated mass effect (Figure 1), which suggested a diagnosis of hyperglycemic hemichorea-hemiballism (HCHB). The patient was started on insulin, and few hours following glucose correction there was great improvement in his mental status and a decrease in involuntary movements. On an unenhanced T1-weighted spin-echo magnetic resonance imaging (MRI) sequence obtained two weeks after initial presentation, there were hyperintense lesions, consistent with hyperglycemic HCHB, located in right putamen. Diffusion-weighted imaging showed no corresponding signal alterations. T2*-weighted imaging demonstrated bilateral punctiform hypointensities in the globus pallidus, which were presumably physiological in nature and did not match the unilateral T1 abnormality (Figure 2). The patient completely recovered his previous cognitive and motor functions after glycemic control, being discharged without sequelae.

Ballistic and choreic movements are characterized by hyperkinetic, random, involuntary movements in the proximal and distal extremities, respectively^(1,2)^. Because they usually occur concomitantly, the term HCHB was created to unify these signs into a clinical syndrome when presented unilaterally. Although HCHB syndrome is secondary to lesions in the basal ganglia, the source of the neuronal damage is controversial, the putative mechanisms including disruption of the blood-brain barrier, decreased thalamic gamma-aminobutyric acid input secondary to anaerobic metabolism, small hemorrhages in the striatal region, hyperviscosity related to hyperglycemia, and Wallerian degeneration of putaminal white matter with protein desiccation^(3,4)^.

Vascular cerebral lesions constitute the most common cause of HCHB^(2)^. Hyperglycemia is considered an important, albeit rare, risk factor for the development of HCHB, which is most commonly seen in elderly female patients with uncontrolled DM. The predominance of Asian patients in the published data suggests an ethnic predisposition. The clinical course tends to vary depending on the patient's glycemic status-the hemiballism and hemichorea usually start together with the hyperglycemia, resolving after its correction^(2,5)^.

Computed tomography findings of hyperglycemic HCHB include unilateral hyperdensity in the basal ganglia contralateral to the affected site. On T1-weighted MRI scans, the most common finding is signal hyperintensity in the caudate nucleus and putamen, usually sparing the internal capsule^(1,6)^. The apparent diffusion coefficient and diffusion-weighted MRI generally indicate restricted diffusion. There is typically no gadolinium enhancement. After glycemic correction, similarly to the clinical findings, such regions tend to return to normal signal intensity.

It is important to highlight the role of susceptibility-weighted imaging (SWI) in differentiating between changes seen in HCHB and areas of calcification or hemorrhage, which represent the most common differential diagnoses. Calcium and blood deposits both generally manifest as hyperintensities on T1-weighted images with corresponding hypointensities on T2*-weighted images and SWI; conversely, HCHB changes tend to present as unilateral hyperintensities on T1-weighted images with no matching changes on T2*-weighted images or SWI^(7,8)^.
